# Assistance to families of children with Autism Spectrum Disorders: Perceptions of the multiprofessional team

**DOI:** 10.1590/1518-8345.5694.3780

**Published:** 2023-03-06

**Authors:** Tassia de Arruda Bonfim, Bianca Cristina Ciccone Giacon-Arruda, Sueli Aparecida Frari Galera, Elen Ferrraz Teston, Francisneide Gomes Pego Do Nascimento, Maria Angélica Marcheti

**Affiliations:** 1 Universidade de São Paulo, Escola de Enfermagem de Ribeirão Preto, PAHO/WHO Collaborating Centre for Nursing Research Development, Ribeirão Preto, SP, Brazil.; 2 Universidade Federal de Mato Grosso do Sul, Instituto Integrado de Saúde, Campo Grande, MS, Brazil.; 3 Scholarship holder at the Conselho Nacional de Desenvolvimento Científico e Tecnológico (CNPq), Brazil.; 4 Escola Paulista de Enfermagem, São Paulo, SP, Brazil.

**Keywords:** Autism Spectrum Disorder, Family, Child, Delivery of Health Care, Health Personnel, Healthcare Models, Transtornos do Espectro Autista, Família, Criança, Atenção à Saúde, Pessoal de Saúde, Modelos de Assistência à Saúde, Trastorno del Espectro Autista, Familia, Niño, Atención de la Salud, Personal de Salud, Modelos de Atención de la Salud

## Abstract

**Objective:**

to synthesize the care provided by health professionals, at different care levels, to the families of children with Autism Spectrum Disorders.

**Method:**

a qualitative study, based on the Family-Centered Care philosophical theoretical framework and developed with 22 professionals from three multidisciplinary teams from the Health Care Network of a municipality in the state of Mato Grosso do Sul, Brazil. The data were collected through two focus groups with each team, organized with the support of the Atlas.ti 8 Qualitative Data Analysis^®^ software and submitted to Thematic Content Analysis.

**Results:**

the findings show actions centered on specific situations, especially on the demands and needs arising from the child’s care and atypical behavior. Influencing factors for family care, such as work overload and little professional experience, show the weakness of multiprofessional care and the invisibility of the family as a care unit.

**Conclusion:**

the need is highlighted to review functioning of the network for the multiprofessional care of children and their families and how such network is organized. It is recommended to offer permanent education actions that contribute to the qualification of multiprofessional teams in the care of families of children in the autism spectrum.

## Resumo

**Objetivo:**

sintetizar o cuidado prestado por profissionais de saúde, nos diferentes níveis de atenção, às famílias de crianças com Transtornos do Espectro Autista.

**Método:**

estudo qualitativo, baseado no referencial teórico filosófico do Cuidado Centrado na Família, desenvolvido com 22 profissionais de três equipes multidisciplinares de serviços da Rede de Atenção à Saúde de um município do estado de Mato Grosso do Sul, Brasil. Os dados foram coletados por meio de dois grupos focais com cada equipe, organizados com apoio do *software Atlas.ti 8 Qualitative Data Analysis* e submetidos à Análise Temática de Conteúdo.

**Resultados:**

as descobertas mostram ações centradas em situações pontuais, principalmente nas demandas e necessidades advindas do cuidado da criança e de seu comportamento atípico. Fatores influenciadores para o cuidado à família, como a sobrecarga de trabalho e a pouca experiência profissional, evidenciam a fragilidade da assistência multiprofissional e a invisibilidade da família enquanto unidade de cuidado.

**Conclusão:**

destaca-se a necessidade de rever o funcionamento e modo como a rede para o cuidado multiprofissional da criança e sua família está organizada. Recomenda-se a oferta de ações de educação permanente que contribuam com a qualificação das equipes multiprofissionais no cuidado às famílias de crianças no espectro do autismo.

## Resumen

**Objetivo:**

resumir la atención que brindan los profesionales de la salud, en los diferentes niveles de atención, a las familias de niños con Trastornos del Espectro Autista.

**Método:**

estudio cualitativo, basado en el marco teórico filosófico de la Atención Centrada en la Familia, desarrollado con 22 profesionales de tres equipos multidisciplinarios de servicios de la Red de Atención de la Salud en un municipio del estado de Mato Grosso do Sul, Brasil. Los datos fueron recolectados a través de dos grupos focales con cada equipo y organizados con la ayuda del *software Atlas.ti 8 Qualitative Data Analysis* ^®^ y sometidos al Análisis de Contenido Temático.

**Resultados:**

los hallazgos muestran acciones enfocadas en situaciones específicas, principalmente en las demandas y necesidades que genera el cuidado del niño y su comportamiento atípico. Factores que influyen en la atención a la familia, como la sobrecarga de trabajo y la poca experiencia profesional, revelan las falencias que tiene la asistencia multidisciplinaria y la invisibilidad de la familia como unidad de atención.

**Conclusión:**

es necesario examinar el funcionamiento y la forma en que está organizada la red para la atención multidisciplinaria de los niños y sus familias. Se recomienda implementar acciones de educación continua que contribuyan a la capacitación de los equipos multidisciplinarios para la atención a las familias de niños con espectro autista.

**Highlights**

(1) Professional practice focused on specific care actions for children with autism.

(2) Care for families through listening, welcoming and Singular Therapeutic Project.

(3) Nurses as links in the multiprofessional team.

(4) Family care fragmentation and weakness in the articulation of the HCN services.

(5) Need for training of the professional team in all health care levels.

## Introduction

The complexity of Autism Spectrum Disorder (ASD) and its manifestations in early childhood, such as communication impairment, social interaction and repetitive behaviors, generate specific care demands for families. Such demands require time, energy and almost exclusive dedication to the child and, sometimes, designation of a family member as the main caregiver, which leads to renouncing to different aspects of personal or professional life to provide the best care possible to the child^1^.

These families are subjected to parental stress due to financial problems, anxiety related to the child’s future, lack of social support, the different implications imposed by the health condition on the group and the increase in care burden, especially in the mothers^2^.

Diverse evidence shows that social support, peer support and hope are strategies that help alleviate these families’ distress^3^ ^-^ ^5^. These strategies have the potential to help them cope with the needs of children with ASD because they favor emotional support and information exchange, promote family well-being, contribute to strengthening family functioning, and relieve anxiety and collective stress. They also provide an optimistic perspective of the future in coping with the condition and in adapting in periods of crisis^3^ ^-^ ^6^.

In Brazil, the Psychosocial Care Network (*Rede de Atenção Psicossocial*, RAPS) proposes guaranteeing access to and quality of the services, from the perspective of comprehensive and multiprofessional care, with a focus on territorial and community-based services, and with the social participation of users and family members. As a Public Policy, the RAPS provides guidelines for the multiprofessional team to act under interdisciplinary logic and ensure inclusion of the family in care planning, aiming to provide articulation and integration of the services in the different points of the network^7^.

In addition to that, the National Policy for the Protection of the Rights of Persons with ASD, enacted in 2012, established that children with ASD and their families should have access to health services, diagnostic processes and multiprofessional care. It determines that actions are carried out that promote qualification and articulation of the professional actions and services to provide adequate health care to these children, ensuring comprehensive care in the scope of primary, secondary and tertiary care^8^.

However, the complexity and demands of care for children with ASD and their families require knowledge, skills and professional attitudes, as well as a qualified and integrated practice, in order to promote an approach centered on the needs experienced by this population segment^1^ ^,^ ^9^.

The national and international literature indicates occurrence of challenges and barriers for health professionals to initiate and maintain health care for this population group. Health professionals’ lack of knowledge and unpreparedness to offer care actions to children with ASD and their families are factors related to this reality, which exerts impacts on care quality and delays access of children and their families to specialized health services^9^ ^-^ ^11^.

These challenges and barriers experienced by families of children with ASD are related to the assistance, support and care offered by health professionals^9^ ^-^ ^10^ ^,^ ^12^. They permeate different moments of the family path, such as the itinerary for diagnosis, the lack of information in the approach by the professionals and services, ineffective communication between health team and family, the family members’ difficulties accessing services, and the lack of support after diagnosis^9^ ^-^ ^13^.

The aforementioned factors turn the search for informal support networks and family hope into a phenomenon experienced by the families, sometimes without participation of the health professionals^14^.

Therefore, as access, care and therapies of these children and their families go through the health professionals belonging to the RAPS, it is necessary to know how they have promoted and guaranteed care to this population^8^.

Thus, the question is as follows: How have the families of children with ASD been assisted by health professionals in the different services of the health care network? Which care actions do the professionals direct to them? This study focuses on the need to explore and synthesize the perception of the multiprofessional team about the care they offer to the families of children with ASD at different health care levels.

The objective of this study was to synthesize the care provided by health professional, at the different care levels, to the families of children with ASD.

## Method

### Study type

An exploratory and descriptive research study with a qualitative approach, based on the Family-Centered Care (FCC) philosophical theoretical framework, whose theoretical assumptions are as follows: dignity and respect, shared information, participation and collaboration^15^.

### Data collection locus

The study was developed in three services of the Health Care Network (HCN) of a capital city from the Brazilian Midwest region. One health service from each care level was included. Thus, choice of loci was for convenience, as follows: a Family Health Unit (FHU), the Child and Youth Psychosocial Care Center (*Centro de Atenção Psicossocial Infanto-Juvenil*, CAPSI) and a Pediatrics ward of a University Hospital (UH).

### Selection criteria

Health professionals with a minimum experience of one month at the workplace were included. This minimum experience time was adopted because, in previous surveys conducted by the research group, turnover of professionals linked to primary and secondary care services was identified due to the organization of the work process of the services. However, during data collection, we identified that the participants’ minimum time of professional activity was 4 months, with a maximum of 17 years.

Those who were on leave or vacation at the beginning of data collection (first focus group) were excluded, as there was a requirement to have taken part in the first group for the other meetings.

### Participants

The study population was defined considering all health professionals belonging to the three health teams. Thus, the participants were all the health service professionals who met the selection criteria and agreed to take part after presenting the research and making the invitation.

### Data collection

The focus group technique was used^16^ for data collection. Two focus groups were held with each service. The first aimed at collecting diverse information and a script prepared by the researchers was used for its conduction, with the following guiding questions: “Have you been caring for children with autism or a similar diagnosis?” and “Describe your experience in caring for these children and their families”. Ancillary questions to deepen the data were also asked, such as the following: “Regarding the families of these children, how have you taken care of them? Do you identify any barriers/difficulties in this care you provided? The second focus group aimed at deepening and validating the content of the initial analysis.

### Procedures for data collection

An individual meeting with each service manager to start data collection was held with the objective of presenting the study and requesting a first meeting with health professionals to make the research invitation to potential participants and scheduling the first focus group.

This first meeting was held with the FHU team, in which 15 professionals took part, as well as with the CAPSi team, with the participation of nine professionals. After the meeting with the responsible manager, at the UH it was requested that the invitation should be made by telephone to the nine professionals of the team who met the selection criteria. 

Both focus groups in each service had been scheduled. The first focus group, in each of the services, was conducted by three researchers: the main researcher (MSc level), in the role of moderator, and two researchers with a PhD (observer and rapporteur); all with previous knowledge in qualitative studies, in the research theme and in conducting focus groups. Eight professionals took part in the GHU: seven in the CAPSi and eight in the UH. 

The second group was conducted by two researchers, one with a PhD and the main researcher. The eight professionals who were included in the first group took part in the FHU. In the CAPSi, four of the seven participants in the first group took part, two did not participate due to work-related requirements on such day, and one for having been transferred to another service. In the UH, of the seven participants in the first group, five professionals took part in the second, and two were unable to attend due to demands of the service on that day.

In the first focus group, a questionnaire with the professionals’ social and labor characteristics was applied in order to characterize the team. There was no pilot test for any instruments; however, they were prepared by the researchers based on previous studies of the group and diverse scientific evidence on the topic.

Both focus groups with each of all three services were held from April to November 2019, in person and in a private room offered by the service. They were recorded on digital audio media. In the FHU, the first focal group lasted 38’ and the second, 33’. In the CAPSi, the first lasted 60’ and the second, 56’. In the UH, they lasted 60’ and 53’, respectively. The research team had no prior working or teaching relationship with the study participants.

### Data treatment and analysis

The first analysis was carried out in detail by the main researcher, and the narratives were transcribed in full and submitted to thematic content analysis^17^. The following stages were performed: 1. Transcription of the interviews, 2. In-depth reading and identification of excerpts considered as indicators of ideas or concepts. Deepening the narratives and capturing the idea, 3. Development of a set of codes (words or short sentences) with the general representation of the narratives; and 4. Association of the sets of codes that had similarities and could comprise initial topics, grouping the excerpts with similar ideas.

After this first moment, a second reading and analysis were carried out, independently, by another two researchers who participated in the collection process. This stage aimed at identifying similarities and divergences in the narratives between the different groups. After the data had been coded and analyzed, a final reading of the transcripts and analysis was carried out in the Atlas.ti 8 Qualitative Data Analysis^®^ software, which assisted in the systematized organization of the codes initially collected.

The participants from the second focus group validated the findings, contributing to deepening and understanding the topics derived from the codes and to the discussion on data saturation. To present the data to the participants, Power Point was used with the categories and topics identified in the first focus group meeting. On this occasion, the participants discussed the topics, which further elucidated the perception of the care provided by the team and closure of the collection stage. The transcripts of this meeting were not returned to the participants.

### Ethical aspects

This study was approved by the Human Research Ethics Committee of the Federal University of Mato Grosso do Sul and developed in accordance with Resolution No. 466/12. The participants were informed about the research and signed the consent form. The statements were identified by the acronyms of the services (FHU, CAPSi and UH) and respective meeting number (1 or 2). 

## Results

A total of 22 health professionals participated in this study: three nurses, two physicians, five community health workers, two social workers, a dentist, two speech therapists, a physiotherapist, a mental health caregiver, three psychologists, an occupational therapist and a health service assistant. Two central topics emerged from the analysis of the narratives: “The professionals’ practice in the care of families of children with ASD” and “Factors that influence care”.

### The professionals’ practice in the care of families of children with ASD

In the health professionals’ experience, the family of a child with ASD has needs related to the demands generated by care, as well as by changes in family dynamics and in the routine of its members.

In Primary Health Care (PHC), the professionals reported believing that the family needs to accept the ASD diagnosis and that psychological support would contribute to reduce denial. *Need for acceptance, someone who accompanies, who is there all the time helping to try to reduce this denial. Psychological support, really.* (FHU 1)

For the secondary care team, the challenges faced, the demands, the time devoted to the child, the changes in family dynamics and social isolation are configured in a challenging context experienced by the families, which requires the team members’ attention. They indicated recognizing that anxiety related to the child’s recovery process permeates the family relationships. *You realize it’s a very big yearning: - When will he get better so I can have a life? In the sense of working, of being productive. [...]. This is a major challenge, because caring for that child requires a lot of energy, time, dedication, so that mothers, fathers and the family can see the child developing. They need to dedicate.* (CAPSi 1)

In hospital care, the professionals reported believing that the families need to be heard and welcomed. They consider the hospital environment stressful, with the potential to cause distress or potentiate already existing emotional issues. They indicated the need for an empathic look and to be prepared for the care of the families. *Need to be heard, need to be welcomed. Even in the conversation circle we focused on that part, welcoming. We have to put ourselves in her place. Sometimes she’s stressed, we need to understand, this empathic look.* (UH 1)

At all levels, the health care practice for these families involved listening, welcoming, conversation circles, home visits, guidelines according to the families’ demands and referrals to specialists. The secondary care service indicated to carry out the welcoming, evaluation and elaboration of the Singular Therapeutic Project (STP). *The guidelines are more in accordance with what they say and we notice, if you see that you’re having personal difficulties looking for a social worker, looking for a psychologist, something like that.* (FHU 2) *[...] everyone, nursing technicians, nurse, psychologist, physiotherapist. Multiprofessional team, all who do the welcoming part! The practice is in charge of everyone, because we can’t do this listening and define completely. We always have to argue. This evaluation is the STP, recommended by the Ministry of Health.* (CAPSi 2)

The professionals reported making referrals or requesting evaluation as a care strategy for the family’s needs, especially in the social and emotional aspects, or with difficulties dealing with atypical behaviors. *You have to guide and forward, depending on the need. If it’s social assistance, if they receive the benefits, we only advise ‘Go to the unit, which has a social worker, and she forwards’.* (FHU 1) *[...] when complicated, atypical behaviors come up, it’s what triggers social work, as if it were an attitude that the mother is not taking care of this child, or with family issues and asks for an evaluation.* (UH 1)

In secondary and tertiary care, another care strategy for the families is the conversation circles for allowing welcoming and listening, as well as a space for the family to expose anxieties and discuss specific demands. The professionals recognized that quality of the service improves when the family has a welcoming space. *I think that quality of the service can improve. You listening, welcoming. I have already seen inside the conversation circle totally distressed families who talk a little, you listen and come out much better, more relieved.* (UH 1)

However, the tertiary care team indicated focusing the attention on the children and their acute conditions at the time of hospitalization, which implies that the professionals do not listen to the family members. In secondary care, the professional care focus is also centered on the children, with actions aimed at the evolution of their development potential. *I think the family is little heard, little listened. Not very welcomed, because the focus is very much on the patient, on the physical disease. We don’t know what the Mom brings, what she has.* (UH 1)*; [...] what changes with this care that we do here, it’s available to families, it’s this view that the child doesn’t have only difficulties, and that within what is possible like him/her, then it’s possible.* (CAPSi 1)

The use of matrix support to assist families in emergency cases was reported by secondary level professionals. However, the team reported difficulties carrying it out because the service covers the entire health territory of the municipality, which implies a delay in performing and weakened assistance. *We need to do the matrix support to make the home visit, which is also a way to support the family or offer this help, which in their territory many things are missing. Sometimes we can’t, so an emergency case goes there, we need to make the visit, follow up, I go there. But we can’t have this as an agenda, because I take care of a large territory.* (CAPSi 1)

In primary care, home visits were highlighted as a care strategy for the families. However, due to the work demands, the priorities of the visits are the families of individuals with a severe and disabling chronic condition, who depend exclusively on the primary care level. *There are some that have more needs, to which we need to provide more priority. These (families of children with ASD) are already being assisted, I need to assist those who have no one. I have to give preferences to them.* (FHU 2)

The primary care team reported difficulties bonding with the families. For them, they do not often access the unit because they are assisted without specialized institutions and consider that the unit lacks adequate resources for their needs. *It was said the other time that it’s just more to refer, there’s not much to do here (unit), because they (family of the child with ASD) have support from the CAPS, APAE, I think this link bond with the hypertensive, diabetic patient, for some cases, happens because here they have what they need most.* (FHU 2)

### Factors that influence family care

Work overload was identified as a factor that hinders family care at different health care levels. In primary care, it was related to the organization of the work process and care centered on the health indicators recommended for PHC.

In secondary care, to the high demand for services, lack of sufficient professionals to meet the number of inhabitants per region, and to organization and functioning of the RAPS. The work overload and demand for procedures, especially of the Nursing team in the hospital context, was indicated as a factor that hinders extended care to the family. *The team is a little small. It affects, it’s complicated, because it’s just one CAPSi for the whole city.* (CAPS 2) *[...] some routines of the day also tie the work, we are often so involved with that routine, technical, administrative. We also have the administrative part, it’s part of the job, it’s not just dealing with the family and the child.* (UH 1)

In the context of tertiary care, there is the team’s tendency to label the family members when they try to learn about the care provided to the child. The professionals referred to this judgment as one of the barriers to family care. *If they (family) want to know what is being done with their child, then they (some team professionals) complain! Because you’re distrusting the work done, that’s not so, that’s not how it works. Or, this is not my role (talking to the family), it’s not my function, there’s nothing to do. [...]. A mother comes who is a little more of a psychiatric patient, a little agitated, really psychiatric, then it becomes total prejudice. The crazy mother, oh there, the crazy mother!* (UH 1)

In hospital care, the institution’s support for the development of actions and activities regarding family care, better communication between teams and definition of the roles of each professional were perceived as facilitating factors. They considered Nursing as the link between all the team members, even with work overload. *It’s defining the roles, the team getting to know each other, how far each person can act. Because Nursing ends up overwhelmed. - In fact, it becomes a link across all the teams (everyone agrees). [...] the team feels supported, even by the institution. Because many times you want to do something, - Ah! But the institution doesn’t give you this support, it seems that you’re doing something isolated.* (UH 1)

In secondary care, the public policies that support health care were identified as ineffective, making it difficult to care for the families. This situation exerts impacts on correct functioning of the HCN, weakens the services, access and follow-up by the population, and discourages the team. *What discourages a little is maybe that we swim and die on the beach. Today the service has a significant demand and a reduced team, and at a certain moment we realize that it affects. To start to get frustrated and sick.* (CAPSi 1)

Tertiary care professionals find it difficult to refer families for lack of knowledge of the network while PHC professionals find difficulties monitoring them in the territory after they access the specialized service. *Do you have a place to refer it to the network? The counter-referral. - It’s a difficulty - isn’t it everyone who has this view where I’m going to refer? [...] because the autism diagnosis is not so easy. So, when some of these changes come up, we end up referring them (referral)... To a psychiatrist; and when they close the diagnosis there, they don’t come back to us anymore.* (FHU 1)

Development and implementation of protocols that indicate the flow and functioning of the care network for the care of children with ASD and their families were indicated as a strategy to deal with the difficulty identified. *There could be a care protocol. What to do during hospitalization and after discharge? Not just with autism, but children with Down, Cerebral Palsy. [Indicates two local institutions]! Which we also don’t know how to access.* (UH 1)

## Discussion

The findings of this study allowed synthesizing that the health care practice by multiprofessional teams provided to the families of children with ASD is carried out through listening and welcoming, with guidelines according to the children’s demands and the care provided to them. However, this practice is still centered on specific situations, mainly on the demands and needs arising from the children’s care and atypical behavior.

Punctual and child-centered care indicates the weakness for including the family in the care process. Therefore, planning and executing actions such as the STP are paths to be followed, as they enable the family to perceive their inclusion in the care provided and in the relationships established with the professionals as fundamental for the children to progress in the therapies^18^.

Use of the STP and matrix support by secondary care professionals was also considered as a family care mechanism by the participants; in addition to referrals to other professionals or special education specialists in the primary- and tertiary-level services. However, assistance cannot be limited to a routine of referrals to specialists and prescriptions and dispensing drugs in PHC^19^.

It should be noted that care limited to referrals is sometimes due to the professionals’ difficulty understanding their role and the situation, as well as in bonding with the service users. A study carried out with professionals from a Family Health Strategy (FHS) unit showed that they can understand the users’ mental health needs, although they have difficulties bonding with these individuals, in qualified listening, in elaborating care proposals and in maintaining their follow-up^20^.

Regarding the families’ access difficulty reported by the PHC professionals and related to social isolation of the family or to its judgment, it is noted that this is sometimes due to discrimination attitudes by the health professionals themselves, which causes mutual distancing^20^. Unwelcoming stances, lack of guidelines, blaming the family and judging the family as a rival are attitudes that the professionals can assume due to lack of training^21^ ^-^ ^22^.

This stance hinders professional-family communication, bonding and trust, and enhances negative experiences, resulting in low care demand from families^22^. Therefore, it is necessary that the health teams are sensitized and trained for care, as knowledge contributes to changing the subjects’ perception, reducing the stigma related to mental disorders, as well as to greater involvement of the professional with the family’s demands^20^ ^,^ ^23^.

The conversation circles, visits and guidelines were referred to by the participants as family care actions. However, they do not consider the family as part of the care provided and do not contribute to the family participating in planning of the child’s care and receiving shared information, violating the assumptions of the FCC philosophy^15^.

In this study, restrictive and facilitating factors that influence the teams’ practice were identified, such as the overload identified at all health care levels. Little collaboration work, excessive demands, impaired communication among the team members and unsuitable management exert an influence on the professionals’ dissatisfaction with the follow-up of work in care^24^. Inadequate conditions in the work environment related to financial, human and material resources imply attention to the users and families cared for. In addition, these conditions impose consequences on the professionals, who describe feeling of devaluation, discouragement with the actions in the service, and repercussions for mental health^25^.

Regarding operation of the RAPS, elaboration of the STP and matrix support was highlighted as an important tool. However, high care demand and the territory covered hinder their execution. Therefore, actions are required to promote the implementation of matrix support, which contributes to articulation of the HCN, in the integration and co-responsibility between primary and secondary care. There is also a need to work with the conflicting relationships between the different services involved, which implies organizing the actions offered by them for good quality mental health care for the individual, the family and the community^26^.

In addition, the data showed the need to review the functioning of the care network for the care of children with ASD and their families and how it is organized, so as to favor not only the assistance provided o this population segment, but also to the family. Collaboration, interdisciplinary and intersectoral work, inclusion of the Family Health Support Center, and training of health professionals are strategies that can reduce the existing problems^18^ ^,^ ^27^.

A study on children’s mental health in PHC also showed lack of knowledge and involvement of the professionals on this topic, making permanent and continuous education actions necessary, in order to improve qualification of the professionals for good quality mental health care for children and families^28^.

Thus, it is necessary that health professionals understand the family as a care unit and dedicate themselves to support, encourage and assist in the decision-making processes necessary for the well-being of children and family members. Implementation of FCC can help health services put into practice care that values the family and guarantees dignity and respect, appreciating history, beliefs and values^15^. Its use favors the user’s perception on the quality of the care received and contributes to supporting the family during the diagnostic process experienced by the parents^11^.

However, there is still a gap in the professionals’ theoretical knowledge about FCC that influences the care context and prevents them from progressing in their involvement with the family. In this sense, strategies that favor the use of theoretical models based on the systemic perspective of Family Nursing contribute for nurses to establish therapeutic conversations with the family^29^ ^-^ ^31^.

Indicated as a link of the multiprofessional team, Nursing can contribute to the use of evaluation and intervention models and help other professionals from the team to interact with the families. The Calgary Family Assessment and Intervention Model (CFAIM) and the Family Intervention Program (FIP) are systematized structures that help nurses work with the families and evaluate and propose interventions aimed at meeting their needs, valuing their strengths and resilience in the face of the disease process^29^ ^,^ ^32^.

It is suggested to carry out studies that address the perception of families of children with mental disorders in access to the RAPS services, as the PHC team considered social isolation of the family (due to the child’s condition) as a factor that hinders access to them. This finding needs to be validated considering the family members’ perception.

As limitations, we can mention the view of a single health team for each care level. Thus, there is a need to invest in expanded research studies for other teams of all three care levels that include the family’s perspective, in order to validate the results and expand them, so as to improve the knowledge adaptation and transfer process.

The results presented have implications in the care context, as they enable transformations in the clinical practice, highlighting the need to approach and include the family of children with ASD in health care in order to turn them into care subjects. The identification of weaknesses in articulation of the RAPS services can contribute to intersectoral actions to ensure better articulation of such services.

## Conclusion

This study sought to explore and synthesize the health care provided by health professionals, at all three care levels, to the families of children with ASD, as well as the factors that influence this care. The professionals understand the families’ experiences and needs. However, in primary and tertiary care, care is focused on the child’s condition and the family is not recognized as a care unit. In secondary care, organizational issues inherent to the service routine hinder more constant care of the family by the professionals.

Knowledge of the factors related to care allows for a reflection on the challenges in health care that has been offered and evidences the fragility of care and the invisibility of families of children with ASD. In addition, it shows that some practices can be modified to enable advances in care quality.

It is recommended that training and permanent education actions be offered, through knowledge transfer. This strategy will enable a qualified and prepared team to take care of these families, to understand them as a care unit and in their needs, and to articulate the RAPS services.
